# Evaluation of Humoral and Cell-Mediated Immunity Induced by Quadrivalent Influenza Vaccine in Pre-COVID-19 Japan

**DOI:** 10.3390/v17050626

**Published:** 2025-04-26

**Authors:** Naruhito Otani, Toshiomi Okuno, Kumiko Yamada, Toshie Tsuchida, Kaori Ishikawa, Kaoru Ichiki, Takashi Ueda, Yoshio Takesue, Satoshi Higasa, Kazuhiko Nakajima

**Affiliations:** 1 Department of Infection Control and Prevention, Hyogo Medical University, Nishinomiya 663-8501, Hyogo, Japan; 2 Department of Public Health, Hyogo Medical University, Nishinomiya 663-8501, Hyogo, Japan; yamakumi@hyo-med.ac.jp (K.Y.); tsuchida@hyo-med.ac.jp (T.T.); i-kaori@hyo-med.ac.jp (K.I.); ichiki@hyo-med.ac.jp (K.I.); taka76@hyo-med.ac.jp (T.U.); takesuey@hyo-med.ac.jp (Y.T.); nakajima@hyo-med.ac.jp (K.N.); 3 Department of Microbiology, Hyogo Medical University, Nishinomiya 663-8501, Hyogo, Japan; tmokuno@hyo-med.ac.jp; 4 Department of Respiratory Medicine and Hematology, Hyogo Medical University, Nishinomiya 663-8501, Hyogo, Japan; parasol@mua.biglobe.ne.jp

**Keywords:** influenza, vaccine, cell-mediated immunity, IFN-γ, hemagglutination inhibition

## Abstract

This study aimed to evaluate the humoral and cell-mediated immune responses induced by an inactivated influenza vaccine (IIV4) during the 2019/2020 influenza season in Japan. We collected blood samples from 25 healthy adults before vaccination, 2 weeks post-vaccination, and 5 months post-vaccination. Humoral and cell-mediated immunities were assessed based on hemagglutination inhibition antibody titers and interferon-γ (IFN-γ) levels, respectively. The geometric mean titer ratio for A/H3N2 exceeded 2.5, meeting the criteria outlined by the European Medicines Agency guidelines; other strains did not achieve similar thresholds. IFN-γ responses indicated significant activation for all strains, with 32–36% of participants exhibiting ≥ 1.5-fold increases. Due to the implementation of infection control measures against COVID-19, influenza activity was not observed during the 2020/2021 and 2021/2022 seasons, potentially altering influenza immunity. Our findings highlight the importance of both humoral and cell-mediated immunity in evaluating vaccine immunogenicity.

## 1. Introduction

Influenza remains a significant global health concern, causing annual epidemics associated with considerable morbidity and mortality [1] and posing a risk of severe complications, particularly in vulnerable populations (such as older adults and individuals with underlying conditions). Vaccination remains the most effective strategy for preventing influenza and its complications [2]. For this study, the 2019/2020 season was selected because it represented the last typical influenza epidemic prior to the implementation of large-scale COVID-19 control measures, making it a valuable reference point for evaluating vaccine-induced immunity in a pre-pandemic context. In Japan, quadrivalent inactivated influenza vaccines (IIV4s) have been widely used since the 2015/2016 season, incorporating two A strains (A/H1N1 and A/H3N2) and two B strains (Victoria and Yamagata lineages) [3,4]. While humoral immunity—assessed through hemagglutination (HA) inhibition (HAI) antibody titers—has been the primary metric for vaccine evaluation, growing evidence suggests that cell-mediated immunity (CMI) also plays a crucial role in protection against influenza [5,6].

The 2019/2020 influenza season in Japan was characterized by the circulation of A/H1N1 and A/H3N2 subtypes, as well as B/Victoria lineage viruses [7]. Immune responses vary depending on the composition of the vaccine strain and individual baseline immunity [8,9]. However, comprehensive assessments of both humoral and cell-mediated immune responses remain limited. The effectiveness of influenza vaccines is influenced by multiple factors, including antigenic match, baseline immunity, and individual immune responses. While humoral immunity—assessed via HAI antibody titers—has traditionally served as the primary indicator of vaccine efficacy, emerging evidence suggests that CMI plays a critical role in long-term protection [10,11]. Studies have shown that T-cell responses, particularly those involving interferon-gamma (IFN-γ) and cytotoxic activity, contribute to viral clearance and immune memory [12]. However, comprehensive evaluations of both humoral and cell-mediated immune responses to influenza vaccines remain limited. While most previous influenza vaccine studies have focused primarily on humoral immunity, recent evidence highlights the importance of CMI in protection and recovery. This study addresses this gap by simultaneously evaluating both humoral and CMI responses, providing a more comprehensive assessment of vaccine-induced immunity.

## 2. Materials and Methods

### 2.1. Study Participants and Vaccines

This study included 25 healthy adult volunteers, comprising 12 males and 13 females (aged 31–58 years), who received the IIV4 between October and December 2019. All participants provided written informed consent and the study was conducted in accordance with the Declaration of Helsinki. The IIV4 used in this study (BIKEN HA; BIKEN, Osaka, Japan) was an egg-derived split vaccine. Each 0.5 mL dose contained 30 μg of hemagglutinin (HA) per strain. The IIV4 (Lot HA18813; BIKEN, Osaka, Japan) was administered subcutaneously at a dose of 0.5 mL. We collected blood samples at three time points: prior to vaccination, 2 weeks post-vaccination, and 5 months post-vaccination. Pre-vaccination samples were collected 1 to 2 weeks before vaccination. All participants had also received the influenza vaccine during the 2018/2019 season. The evaluation included measurements of CMI (assessed through IFN-γ levels at baseline and 2 weeks post-vaccination) and humoral immunity (assessed through HAI titers at all three time points). All participants received the influenza vaccine during the 2019/2020 season. The study was approved by the Ethics Committee of Hyogo Medical University (protocol number: 1592). All participants had also received the influenza vaccine during the 2018/2019 season, which included the same B/Yamagata strain as in 2019/2020.

### 2.2. Antigens

Since the 2015/2016 influenza season, the IIV4 in Japan has included antigens from A/H1N1, A/H3N2, B/Victoria, and B/Yamagata strains. The A/H1N1 and A/H3N2 components had been updated since the 2018/2019 season (Table 1). For the 2019/2020 season, the vaccine contained A/Brisbane/02/2018 (H1N1)pdm09, A/Kansas/14/2017 (H3N2), B/Phuket/3073/2013 (B/Yamagata lineage), and B/Maryland/15/2016 (B/Victoria lineage). Vaccine antigens, including those used in the IFN-γ release assay, were provided by BIKEN.

### 2.3. HAI Antibody Measurement

HAI antibody titers were determined using the vaccine strains. To remove nonspecific inhibitors, serum samples were treated with a receptor-destroying enzyme (Denka Seiken Co., Tokyo, Japan) and subsequently diluted 1:10. The HAI titers were measured using an influenza virus HAI assay (Denka Seiken Co.) with the chicken red blood cells (RBCs) provided in the assay kit. The highest dilution showing complete HAI was recorded as the HAI titer. Titers ≥ 1:10 were considered positive. All the assays were performed by a commercial laboratory (SRL Inc., Tokyo, Japan).

### 2.4. IFN-γ Assay

The IFN-γ assays were conducted as previously described [13]. Whole blood samples (100 µL) were mixed with 100 µL of RPMI 1640 medium containing vaccine antigens; these were prepared at final concentrations of 5 HA units (equivalent to 5 μg HA/mL) and 10 HA units (equivalent to 10 μg HA/mL) per well, respectively, in 96-well microtiter plates. Two antigen concentrations (5 HA and 10 HA units) were used to assess the sensitivity of IFN-γ responses at different levels of stimulation. Responses to each concentration were analyzed separately. This resulted in a total volume of 200 µL per well. All assays were performed in duplicate. IFN-γ levels were measured in duplicate wells for each participant, and the average of the two wells was used for analysis. The plates were incubated at 36.5 °C with 5% CO_2_ for 48 h, after which the supernatants were collected and stored at −80 °C. IFN-γ levels (pg/mL) in the supernatants were measured using an enzyme-linked immunosorbent assay (ELISA; Human IFN-gamma ELISA Kit; Invitrogen, Waltham, MA, USA) following the manufacturer’s instructions. Wells with medium alone served as negative controls, and IFN-γ levels < 4 pg/mL were classified as negative. A 1.5-fold increase in IFN-γ concentration post-vaccination was defined as a positive cell-mediated immune response [13].

### 2.5. Evaluation of Changes in Antibody

The European Medicines Agency guidelines [14] for assessing immune responses to influenza vaccines in individuals aged 18–59 years were applied. According to the guidelines, at least one of the following criteria must be met: (1) ≥70% of participants achieving HAI titers of ≥1:40 post-vaccination; (2) ≥40% of seronegative participants achieving seroconversion (titer ≥ 1:40) or ≥4-fold titer increases in seropositive participants; (3) a geometric mean titer (GMT) ratio (GMTR) > 2.5.

### 2.6. Statistical Analyses

Spearman rank correlations were calculated to assess the correlation between the results of the HAI and IFN-γ assays, with statistical significance set at *p* < 0.05. All statistical analyses were conducted using SPSS version 29 (IBM Corp., Armonk, NY, USA).

## 3. Results

### 3.1. Changes in Antibody Titers Post-Vaccination

#### 3.1.1. Criterion 1: Proportion of Participants with HAI Titers ≥ 1:40

Two weeks post-vaccination, the proportions of participants with HAI titers ≥1:40 were as follows: A/H1N1 (40%), A/H3N2 (60%), B/Yamagata (68%), and B/Victoria (48%). None of the vaccine antigens met the ≥70% threshold specified as a criterion in the guidelines (Table 1).

#### 3.1.2. Criterion 2: Seroconversion or ≥4-Fold Increase in HAI Titers

Two weeks post-vaccination, the proportions of participants who achieved seroconversion or a ≥4-fold increase in HAI titers were as follows: A/H1N1 (0%), A/H3N2 (20%), B/Yamagata (0%), and B/Victoria (4%). None of the vaccine antigens met this criterion (Table 1).

**Figure 1:**
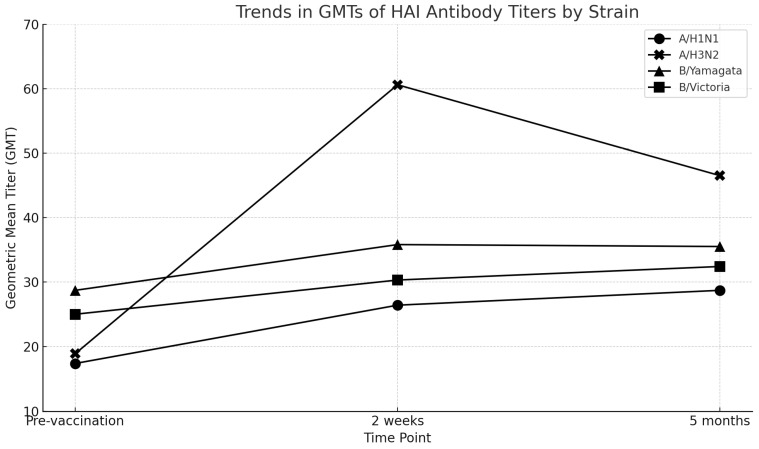
The trends in GMTs for each influenza strain across the three time points: pre-vaccination, 2 weeks post-vaccination, and 5 months post-vaccination. This graphical representation enhances the interpretation of the humoral immune response over time.

Two weeks post-vaccination, the GMTR values were as follows: A/H1N1 (1.5), A/H3N2 (3.2), B/Yamagata (1.2), and B/Victoria (1.2). Only A/H3N2 achieved a GMTR > 2.5 (Figure 1, Table 2).

### 3.2. Changes in CMI (IFN-γ)

The geometric mean concentration (GMC) and GMC ratio (GMCR) of IFN-γ were evaluated using vaccine antigen levels of 5 HA units and 10 HA units, respectively (Table 3). When pre-vaccination GMCs were compared with results from a previous study [15], the values obtained with 5 HA unit antigen levels were similar, indicating that the ELISA kit used in the present study was capable of measuring cellular immunity (IFN-γ) with a smaller amount of antigen than previously used.

Positive responses (≥1.5-fold increase in IFN-γ) were observed in 28% of participants for A/H1N1, 28% for A/H3N2, 36% for B/Yamagata, and 32% for B/Victoria (Table 4).

### 3.3. Relationship Between HAI and CMI Before and After Vaccination

Spearman’s rank correlation analysis was performed to assess the relationship between HAI titers and IFN-γ levels for each influenza strain pre-vaccination and post-vaccination. Pre-vaccination: no significant correlation was observed between HAI titers and IFN-γ levels before vaccination for any of the four influenza strains (*p* > 0.05 for all strains). Post-vaccination: after vaccination, a significant negative correlation was observed between HAI titers and IFN-γ responses for the B/Yamagata strain (r= −0.52, *p* = 0.007), suggesting that individuals with higher baseline humoral immunity exhibited a weaker cell-mediated response. For the A/H1N1 (r = 0.16, *p* = 0.446), A/H3N2 (r = 0.14, *p* = 0.496), and B/Victoria (r = –0.14, *p* = 0.503) strains, no significant correlations were observed (*p* > 0.05 for all strains).

## 4. Discussion

The findings of this study provide important insights into the immunogenicity of the IIV4 during the 2019/2020 season. It has previously been found that antibody status was found to vary by age for the four influenza viruses used as vaccine strains in this season [16].

The GMT for A/H3N2 remained elevated 5 months post-vaccination, demonstrating sustained humoral immunity. This result aligns with observations from the 2017/2018 season but differs from the findings of the 2018/2019 season, wherein a decline in GMT was observed [8,17]. These differences may reflect variations in vaccine strain composition, baseline immunity, and external factors, such as influenza exposure. The sustained GMTR exceeding 2.5 for A/H3N2 further supports its potential efficacy, particularly compared with strains that did not meet this threshold [14]. In Japan, these EMA criteria are commonly referenced in the evaluation of influenza vaccines. However, given the limited sample size, this comparison is provided for contextual reference only and not for formal assessment. The durability of the immune response underscores the importance of evaluating year-to-year variations in vaccine performance. These differences may reflect factors such as antigenic variability between strains, prior immunity from previous vaccinations, or differences in the baseline cellular response across influenza subtypes.

In this study, CMI was assessed through IFN-γ responses. A significant proportion of participants (28–36%) exhibited ≥1.5-fold increases in IFN-γ levels for all the tested strains, indicating the robust activation of CMI [13]. This finding underscores the complementary role of CMI alongside humoral immunity in providing broader protection against influenza. Notably, the use of a more sensitive ELISA kit enabled the detection of IFN-γ responses with lower antigen concentrations, suggesting improvements in the assessment of vaccine-induced cellular immunity [15].

In this study, HAI titers negatively correlated with CMI responses for the B/Yamagata strain, with a correlation coefficient of −0.52 (*p* < 0.05), suggesting that individuals with stronger pre-existing humoral immunity may exhibit weak CMI activation after vaccination. The possible mechanisms for this include antigen competition or immune feedback that modulates the magnitude of cellular responses. Further research is needed to clarify whether this phenomenon is specific to the B/Yamagata strain or represents a broader immunological principle. The unchanged B/Yamagata strain from the previous season may have influenced the observed immune responses due to pre-existing immunity, raising the possibility of original antigenic sin. Furthermore, it is noteworthy that no confirmed detections of circulating B/Yamagata lineage viruses have been reported since March 2020, raising the possibility that this lineage may have become extinct. This observation is consistent with the WHO’s recommendation for the 2023–2024 influenza season [18]. Despite these advancements, interindividual variability in CMI responses highlights the need for further research. Factors such as baseline immunity, age, and prior influenza exposure are likely to influence the magnitude of CMI responses and warrant further investigation in larger and more diverse cohorts.

The sustained antibody response observed for A/H3N2 and the activation of CMI for all strains suggest that IIV4 formulations may offer extended protection against influenza. These findings have significant implications for vaccination strategies, particularly for high-risk populations (such as older adults) who may benefit from tailored vaccination schedules or booster doses [9].

We acknowledge that age-related differences in antibody responses could influence vaccine efficacy, particularly in older adults. Previous studies have shown that aging is associated with immunosenescence, which affects both humoral immunity and CMI [10]. The observed negative correlation between HAI titers and IFN-γ responses for the B/Yamagata strain may be more pronounced in older adults, who often have a history of repeated influenza vaccinations, potentially shaping their immune responses differently.

These findings suggest that conventional influenza vaccines may not be equally effective in all age groups. Hence, vaccination strategies for older adults should consider alternative approaches—such as the use of adjuvanted vaccines or high-dose formulations—to enhance both humoral and cellular immune responses in this population. Further studies are needed to optimize influenza vaccination strategies for older adults by incorporating both humoral and cell-mediated immune responses into vaccine evaluation criteria.

While this study provides valuable insights, its limitations include a small sample size and a lack of age stratification. Future studies should include larger and more diverse populations to better understand variability in immune responses. Furthermore, evaluating the combined effects of humoral and CMI responses on clinical outcomes, such as reduced influenza incidence or severity, will be essential. Advances in CMI assessment techniques should also be leveraged to facilitate its widespread application in vaccine evaluation [13,15].

Many influenza vaccines are in use, with variable immunogenicity [19]. Therefore, comparative studies of immunogenicity among different vaccines are necessary. Influenza vaccination reduces the severity of COVID-19 [20]. Thus, the immunological relationship between influenza vaccination and COVID-19 should also be assessed.

The number of influenza cases during the 2024/2025 season has returned to pre-COVID-19 levels [21]. Given the reduced circulation of influenza during the COVID-19 pandemic, which likely resulted in decreased population immunity, assessing immune responses from pre-COVID-19 vaccination remains crucial for understanding baseline immunity levels and improving vaccine strategies.

## 5. Conclusions

This study underscores the critical role of both cell-mediated and humoral immunity in assessing the efficacy of influenza vaccines. The findings contribute to the optimization of vaccination strategies and highlight the need for the continued evaluation of immune responses to ensure effective protection against evolving influenza strains. However, given our limited sample size and single-season scope, the findings should be interpreted as preliminary, and further large-scale studies are needed to confirm these results.

## Figures and Tables

**Table 1:** Influenza vaccine strain composition and summary of serological responses during the 2019/2020 season.

Influenza Vaccine Antigen	2018/2019 Season	2019/2020 Season	HAI ≥ 1:40 (Pre)	HAI ≥ 1:40 (2 w)	HAI ≥ 1:40 (5 m)	≥4-fold Increase (2 w)	≥4-fold Increase (5 m)
A/H1N1	A/Singapore/GP1908/2015(IVR-180)	A/Brisbane/02/2018(IVR-190)	28% (7/25)	40% (10/25)	39% (9/23)	4% (1/25)	0% (0/23)
A/H3N2	A/Singapore/INFMH-16-0019/2016(IVR-186)	A/Kansas/14/2017(X327)	32% (8/25)	60% (15/25)	57% (13/23)	20% (5/25)	17% (4/23)
B/Yamagata lineage	B/Phuket/3073/2013	B/Phuket/3073/2013	60% (15/25)	68% (17/25)	61% (14/23)	0% (0/25)	0% (0/23)
B/Victoria lineage	B/Maryland/15/2016(NYMC BX-69A)	B/Maryland/15/2016(NYMC BX-69A)	44% (11/25)	48% (12/25)	52% (12/23)	4% (1/25)	0% (0/23)

HAI, hemagglutination inhibition.

**Table 2:** HAI antibody titers before and after vaccination.

Influenza Vaccine Antigen	HAI		
	GMT Pre- and Post-Vaccination (GMTR)		
	Pre-Vaccination	2 Weeks	5 Months
A/H1N1	17.4	26.4 (1.5)	28.7 (1.6)
A/H3N2	18.9	60.6 (3.2)	46.5 (2.5)
B/Yamagata lineage	28.7	35.8 (1.2)	35.5 (1.2)
B/Victoria lineage	25.0	30.3 (1.2)	32.4 (1.3)

HAI, hemagglutination inhibition; GMT, geometric mean titer; GMTR, geometric mean titer ratio.

**Table 3:** IFN-γ concentrations (pg/mL) after vaccination.

Influenza Vaccine Antigen	5 HA Units		10 HA Units	
	Pre-Vaccination GMC	2 Weeks GMC (GMCR)	Pre-Vaccination GMC	2 Weeks GMC (GMCR)
A/H1N1	79.4	100.0 (1.3)	134.0	130.0 (1.0)
A/H3N2	71.1	89.3 (1.3)	105.9	117.0 (1.1)
B/Yamagata lineage	75.6	104.0 (1.4)	118.8	144.9 (1.2)
B/Victoria lineage	74.7	95.8 (1.3)	115.7	136.4 (1.2)

HA, hemagglutinin; GMC, geometric mean concentration; GMCR, geometric mean concentration ratio.

**Table 4:** Proportion of GMCRs ≥ 1.5 after vaccination.

Influenza Vaccine Antigen	GMCR ≥ 1.5
	2 Weeks Post-Vaccination
H1N1	28% (7/25)
H3N2	28% (7/25)
B/Yamagata lineage	36% (9/25)
B/Victoria lineage	32% (8/25)

GMCR, geometric mean concentration ratio.

## Data Availability

The raw data supporting the conclusions of this article will be made available by the authors upon request.

## Abbreviations

The following abbreviations are used in this manuscript:

IIV4	Quadrivalent inactivated influenza vaccine
HAI	Hemagglutination inhibition
CMI	Cell-mediated immunity
IFN-γ	Interferon-γ
ELISA	Enzyme-linked immunosorbent assay
GMTR	Geometric mean titer ratio
GMT	Geometric mean titer
HA	Hemagglutinin
GMC	Geometric mean concentration
GMCR	Geometric mean concentration ratio
